# Supplementary figures and images for: The axonal sorting activity of pseudorabies virus Us9 protein depends on the state of neuronal maturation
Source: PLoS Pathog. 2020 Dec 28;16(12):e1008861. doi: 10.1371/journal.ppat.1008861 (PMC7794026; doi:10.1371/journal.ppat.1008861)

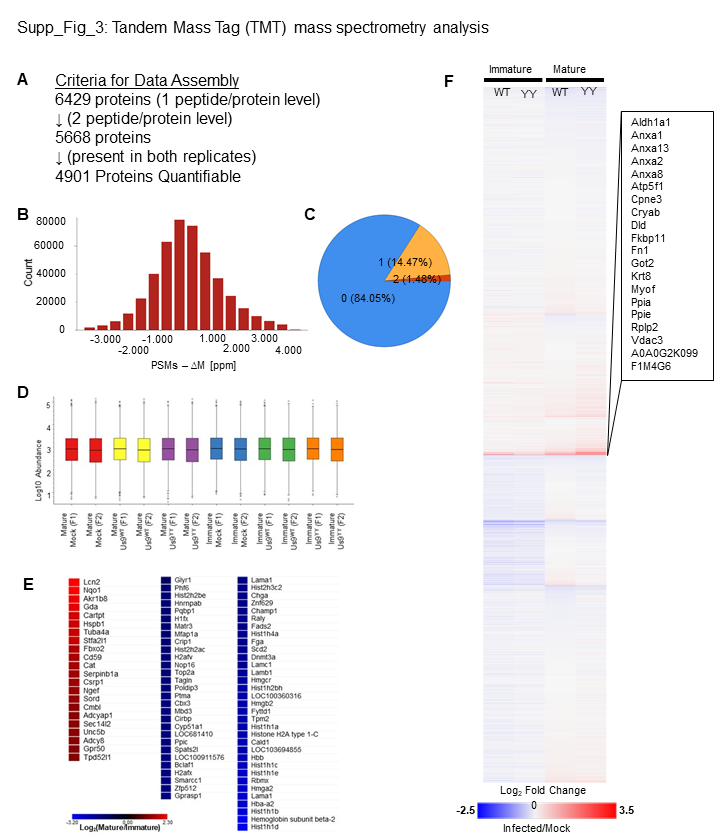

Supplement: S1 Fig — A: Criteria used to filter the data set. B: Mass Accuracy: High mass accuracy centered about 0ppm is reliable. C: Missed Cleavages: Relatively low missed cleavage rate demonstrating thorough trypsin digest of peptides. D: Sample Abundances: Equal abundances across all sample suggests comparable mixing between samples. E: Differences in mock mature versus immature proteome. These proteins were significantly differential by background-based ANOVA analysis and are ordered by abundance levels (higher in Mature on upper left and higher in immature in bottom right). Chromatin organizing proteins are overrepresented here (adjusted P-value <7.68E-10). F: TMT reporter ion values for host proteins: Many proteins express similar levels. A small subset appears enriched in all infection conditions; the individual protein names are listed in the inset. (TIF) [file ppat.1008861.s003.tif]

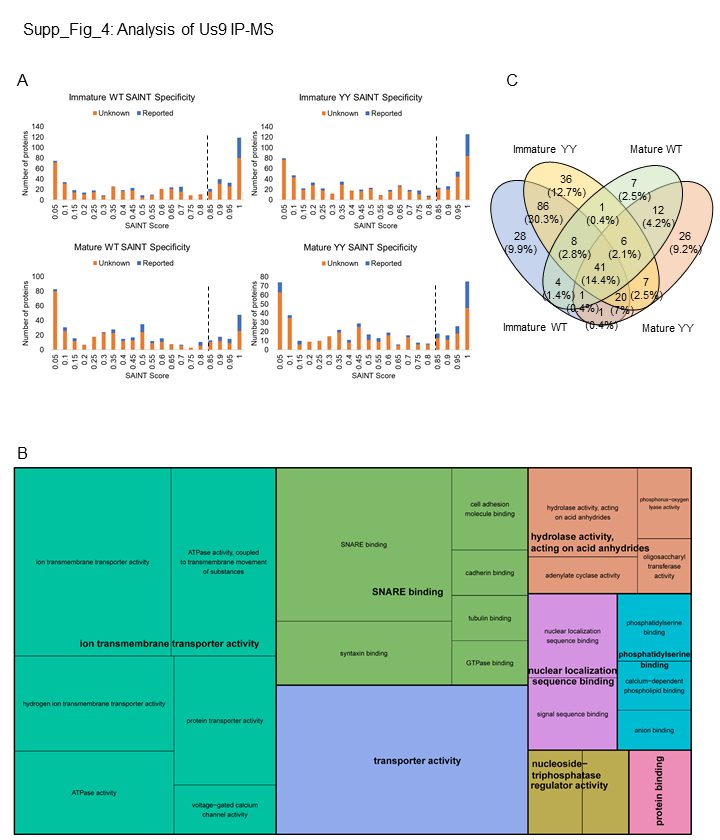

Supplement: S2 Fig — A: SAINT cutoff (dashed line) of ≥0.85 chosen for Us9 baits based on histogram of SAINT scores for novel (orange) and previously reported (blue) interactions. B: Treemap of Enrichment analysis by GO (gene ontology) MF (molecular function) terms. Functionally related categories are grouped by color and boxes are sized by adjusted p-value. Enrichment analysis of all specificity-filtered Us9 interacting proteins shows enrichment in transporter and SNARE binding activity. C: Venn diagram of specificity-filtered proteins in Us9WT and Us9YY IPs highlights that some proteins are found as interactors in both neuronal developmental stages and some are unique to one stage. (TIF) [file ppat.1008861.s004.tif]

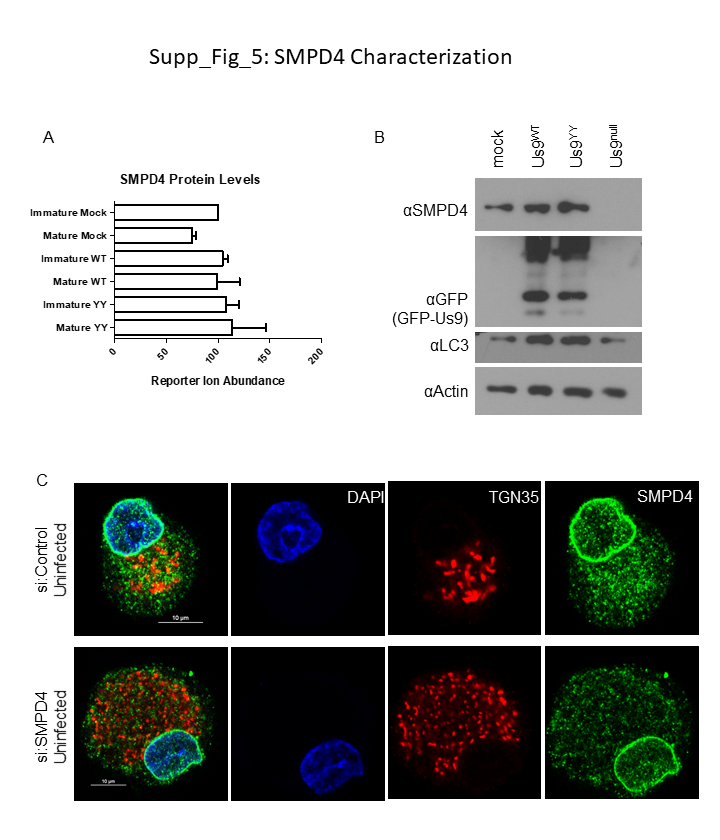

Supplement: S3 Fig — A: SMPD4 protein abundance values from mass-spectrometry. In uninfected/mock dissociated SCGs, SMPD4 is slightly more abundant in mature neurons compared to immature. Infection increases abundance levels compared to mock, but SMPD4 levels do not change between Us9WT or Us9YY infection conditions. B: SMPD4 protein expression is stimulated by Us9. Dissociated SCG neurons were infected with mock (uninfected), Us9WT, Us9YY, or Us9null PRV strains to assay changes in SMPD4 protein expression. At 12hpi, samples were lysed and subject to SDS-PAGE for SMPD4 and Actin proteins. SMPD4 protein expression is the strongest upon Us9WT infection, followed by comparable levels in mock and Us9YY infection, and lowest in Us9null infection. C: SMPD4 localization in uninfected SCG neurons. Confocal microscopy images of dissociated SCG cell body transduced with siRNA against Non-Target control (top) or SMPD4 (bottom) followed by immunofluorescence staining with DAPI, TGN38 for golgi and SMPD4. (TIF) [file ppat.1008861.s005.tif]
